# Composition of *Polygonatum zanlanscianense* Pamp. Steam and Leaf Phenolic Extract and Its Protective Mechanism on t-BHP-Induced Oxidative Damage of HepG2 Cells

**DOI:** 10.3390/molecules28227487

**Published:** 2023-11-08

**Authors:** Shuang Tang, Jin Yong, Jin Yan, Teng Peng, Fei Long, Hulan Chen

**Affiliations:** School of Pharmacy, Chengdu University of Traditional Chinese Medicine, Chengdu 611137, China; shuangt2023@outlook.com (S.T.); yongjin@stu.cdutcm.edu.cn (J.Y.); yjwell@yeah.net (J.Y.); longfei@cdutcm.edu.cn (F.L.); chenhulan@cdutcm.edu.cn (H.C.)

**Keywords:** antioxidant activity, phenol, *Polygonatum zanlanscianense* Pamp., steam and leaf, oxidative stress, UPLC-Q-Obtrip-MS

## Abstract

Plant phenolic compounds have attracted considerable attention because of their health benefits. This study aimed to investigate the composition and antioxidant activity of phenol extracts from *Polygonatum zanlanscianense* Pamp. steam and leaf (PPP). The FTIR, UPLC-Q-Obtrip-MS, and HPLC−DAD methods were used to analyze the composition of PPP, and 20 phenolic compounds were preliminarily identified. Among them, the contents of hyperin, astragalin, and diosmetin levels were the highest. Treatment with PPP can significantly reduce t-BHP-induced cell damage in HepG2 cells, reactive oxygen species (ROS) accumulation, and malondialdehyde (MDA) content. Meanwhile, the superoxide dismutase (SOD), catalase (CAT), glutathione (GSH), and glutathione peroxidase (GSH−Px) activities can be increased. Moreover, PPP enhanced Nrf2 expression, which was consistent with that of heme oxygenase-1 (HO−1), glutamate–cysteine ligase catalytic subunit (GCLC), and NAD(P)H quinone oxidoreductase-1 (NQO1), whereas the expression of Keap1, the Nrf2 inhibitor, was decreased. All findings indicate that PPP can serve as a natural bioactive substance for preventing oxidative stress.

## 1. Introduction

In recent years, numerous studies have shown that reactive oxygen species (ROS) are closely related to human health. Under normal physiological conditions, low-level ROS production is considered to be a signal molecule. Numerous chronic and degenerative disorders, including cancer, cardiovascular diseases, diabetes, neurological diseases, and aging, can be brought on by excessive ROS production [1,2]. Due to their safety, potent antioxidant capacity, and few side effects, natural antioxidants are drawing increasing attention. Because of their detrimental health impacts, synthetic antioxidants have drawn attention in the food industry [3]. Therefore, natural antioxidants are urgently needed to replace artificially synthesized antioxidants.

With the rapid development of the traditional Chinese medicine planting industry, many straw resources will be generated during planting, processing, and production. The accumulation and incineration of large amounts of straw will pollute the ecological environment and cause a massive loss of resources. Therefore, the development and implementation of straw resources can result in the recycling of agricultural resources while additionally greatly raising the earning capacity of producers. Phenolic compounds are receiving increasing attention in straw resources. Some studies [4,5,6,7,8] have revealed that the content of phenolic compounds in straw is higher than that in medicinal parts of plants, and that they have several important biological activities, including antioxidant, antitumor, neuroprotective, and antibacterial activities. The antioxidant biological activity exhibited by plant phenolic extracts can serve as an indirect antioxidant that activates the body’s antioxidant mechanism by regulating the expression of intracellular antioxidant enzymes, thereby maintaining the body’s redox homeostasis [9]. Antioxidant and detoxifying enzymes, including NAD(P)H quinone oxidoreductase-1 (NQO1), superoxide dismutase (SOD), malondialdehyde (MDA), catalase (CAT), glutathione (GSH), glutamate–cysteine ligase catalytic subunit (GCLC), glutathione peroxidase (GSH−Px), and heme oxygenase-1 (HO−1), regulate the endogenous antioxidant system of cells. Under oxidative stress damage, they can eliminate excess ROS from the body. Thus, they play an important role in maintaining intracellular redox homeostasis [10]. Nrf2 refers to nuclear factor E2-related factor 2, which regulates the expression of these antioxidant enzymes. When Nrf2 is activated, it dissociates from the Keap1 protein and transfers from the cytoplasm to the nucleus, binding to the antioxidant response element (ARE) in the nucleus. This induces the expression of downstream antioxidant enzymes and plays a role in maintaining redox homeostasis in the body [11].

*Polygonatum zanlanscianense* Pamp. belongs to the family Polygonatum, widely cultivated in various parts of China [12]. “The Outline of Xinhua Materia Medica” records that “rhizome has the same effect as Polygonatum, which invigorates the spleen, moistens the lung, and kills insects”. “The Dictionary of Traditional Chinese Medicine” records that “the root of *Polygonatum zanlanscianense* Pamp. has long been used to treat lung diseases, palpitations, stomach discomfort, and diabetes”. At present, research on and applications of the chemical components of *Polygonatum zanlanscianense* Pamp. mainly focus on the rhizomes, and the stem and leaf parts, which occupy half of the weight of the entire grass, are often discarded and burned. This not only causes great waste of *Polygonatum zanlanscianense* Pamp. resources, but also causes environmental pollution. The stems and leaves of *Polygonatum zanlanscianense* Pamp. also have significant medicinal value. Previous studies have shown that *Polygonatum zanlanscianense* Pamp. leaves are rich in furans, acids, and phenolic substances, and have strong free radical scavenging ability [13]. However, the composition of the stem and leaf components of *Polygonatum zanlanscianense* Pamp. and its antioxidant mechanism at the cellular level is still unidentified.

Therefore, the aim of this study was to clarify the possible process by which *Polygonatum zanlanscianense* Pamp. phenols exert antioxidant effects. We used an ultrasonic extraction method to extract phenols from the stems and leaves of *Polygonatum zanlanscianense* Pamp. and purified them with macroporous resin. The components of the phenolic extract were analyzed using FTIR, UPLC-Q-Obtrip-MS, and HPLC-DAD. The HepG2 cell line is accessible, representative, stable, shows metabolic activity, and demonstrates ease of detection, so it is often used as an oxidative damage model [14,15]. Therefore, the protective execution capabilities of the phenolic extract on t-BHP-induced oxidative damage was studied utilizing a HepG2 cell system.

## 2. Results

### 2.1. Qualitative and Quantitative Analysis of the Main Phenols in PPP

#### 2.1.1. FTIR Analysis

**Figure 1:**
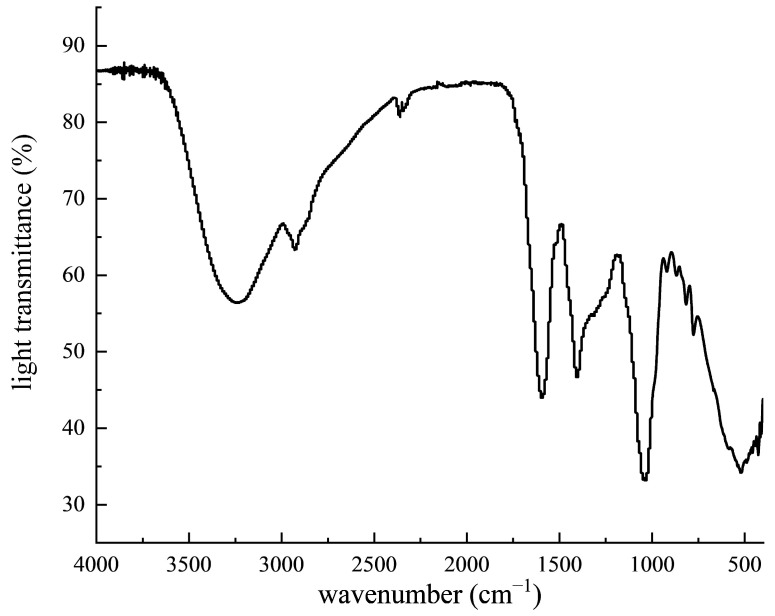
Infrared spectra of PPP.

Figure 1 shows the infrared characteristic spectrum of PPP. The stretching vibration of O-H caused an absorption peak at the wave number of 3408.56 cm^−1^. The C-H stretching vibration of -CH_2_- caused an absorption peak at the wave number of 2918.87 cm^−1^. The stretching vibration of carbonyl C=O caused an absorption peak at the wave number range of 1640.64 cm^−1^. The stretching vibration of aromatic compounds caused an absorption peak at the wave number of 1594.84 cm^−1^. An absorption peak appeared at 1405.37 cm^−1^, indicating the possibility of phenolic hydroxyl groups.

#### 2.1.2. UPLC-Q-Obtrip-MS Analysis

**Figure 2:**
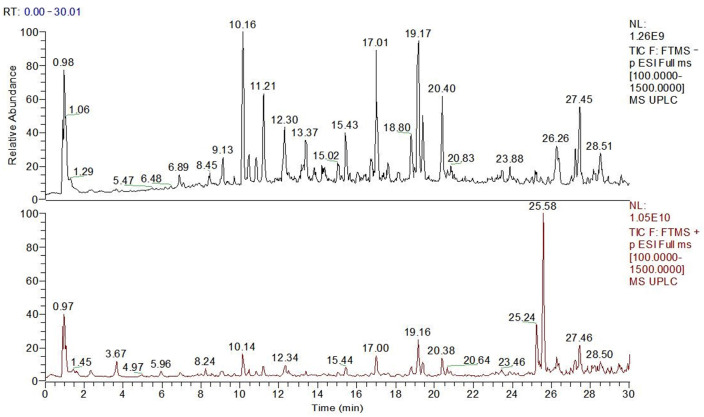
UPLC-Q-Obtrip-MS chromatogram of PPP.

Figure 2 shows the UPLC-Q-Obtrip-MS chromatogram of PPP. There were 20 phenolic compounds identified in this study (Table 1), including 3 phenylpropanoic acid compounds: caffeic acid, chlorogenic acid, and ferulic acid, and 17 flavonoids and their glycosides.

#### 2.1.3. HPLC Analysis

The peak time was determined using a single standard sample, and 12 monomeric phenols were used as a mixed standard sample to determine the composition of the purified phenols using HPLC-DAD. The content of the components with a peak area >5% was determined.

**Figure 3:**
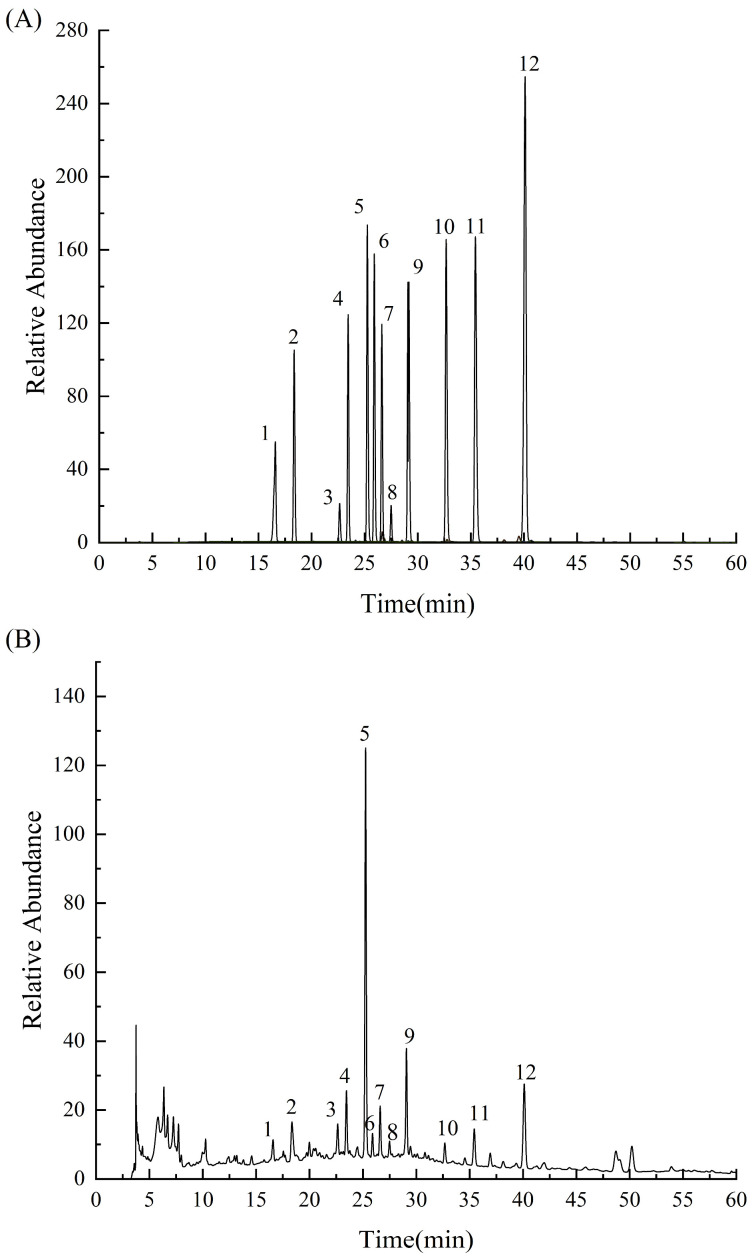
HPLC results of the mixed phenol standard (**A**) and PPP (**B**). Peak1: chlorogenic acid, peak 2: caffeic acid, peak 3: taxifolin, peak 4: ferulic acid, peak 5: hyperin, peak 6: isoquercitrin, peak 7: rutin, peak 8: hesperidin, peak 9: astragalin, peak 10: quercetin, peak 11: luteolin, and peak 12: diosmetin.

Figure 3 shows that the peak time of each peak in the sample was consistent with the time of the standard sample, indicating that PPP contained these 12 phenolic components. Hyperin, astragalin, and diosmetin contents were relatively high at 76.6, 21.4, and 26.4 mg/g, respectively (Table 2).

### 2.2. The Effect of PPP and t-BHP on the Proliferation of HepG2 Cells

**Figure 4:**
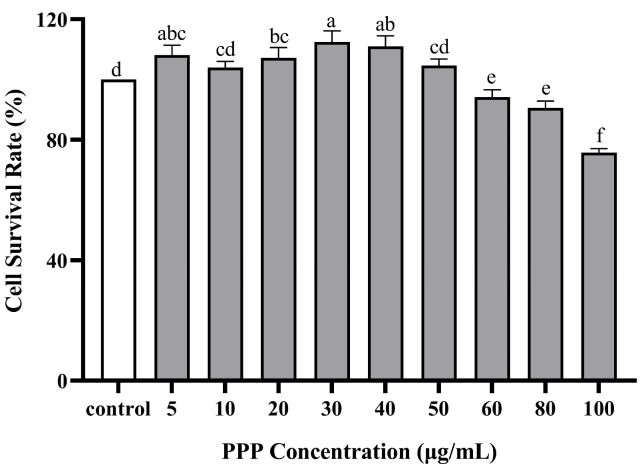
Effect of PPP on the cell viability of HepG2 detected by CCK-8. Those with the same letter in each column indicate no significant difference (*p* < 0.05), and those with different letters indicate significant difference (*p* < 0.05). The farther apart the alphabetical order, the greater the difference.

**Figure 5:**
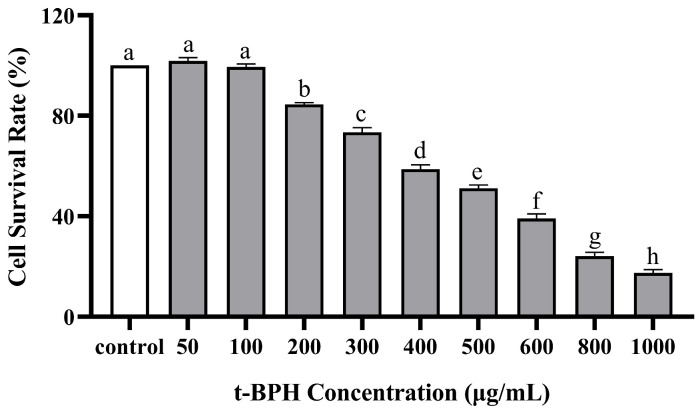
Effect of t-BHP on the cell viability of HepG2 detected by CCK-8. Those with the same letter in each column indicate no significant difference (*p* < 0.05), and those with different letters indicate significant difference (*p* < 0.05). The farther apart the alphabetical order, the greater the difference.

The effects of PPP and t-BHP on the proliferation and toxicity of HepG2 cells were measured using a CCK-8 assay (Figure 4 and Figure 5). Cytotoxicity was investigated in the presence of PPF alone at different concentrations of 0–100 µg/mL. No cell toxicity was observed at 0–50 µg/mL concentrations, and the cell survival rate was >95%. Subsequently, as the PPP concentration increased, the cell survival rate decreased, indicating that when the concentration exceeded 50 µg/mL, it had a certain toxic effect on HepG2 cells. Based on the above results, three concentrations of 10, 30, and 50 µg/mL were selected to study the protective effect of PPP on t-BHP-induced oxidative damage in HepG2 cells, meaning that the PPP group was divided into a high group (HG), medium group (MG), and low group (LG), respectively. Furthermore, when the t-BHP concentration increased from 0 µg/mL to 1000 µg/mL, the cell survival rate significantly decreased dose-dependently. At low concentrations, t-BHP caused less damage to HepG2 cells, whereas excessive concentrations caused irreversible damage to the cells. When the cell survival rate was between 50% and 70%, the cells underwent oxidative damage and had a certain degree of recovery ability. Therefore, a concentration of 500 µg/mL t-BHP intervention for 2 h was used as the modeling condition for subsequent experiments.

### 2.3. The Effect of PPP on the Level of Intracellular ROS Induced by t-BHP

**Figure 6:**
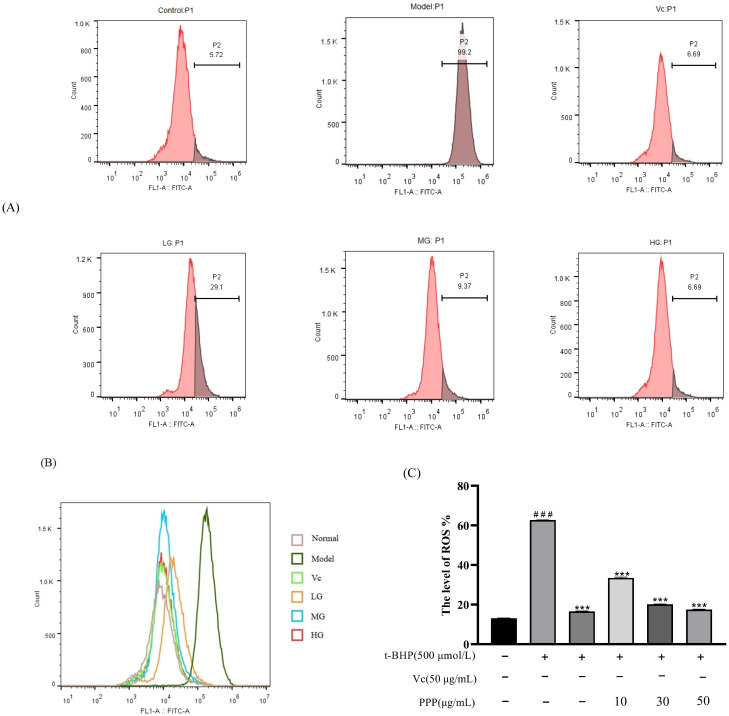
The average fluorescence intensity was measured using flow cytometry (**A**,**B**). Statistical analysis of ROS generation (**C**). Control group was used as a positive control. Data are presented as mean ± SD. *n* = 3, ^###^ *p* < 0.001 vs. control group, *** *p* < 0.001 vs. model group.

ROS in normal cells are maintained at a certain level, and when cells are stimulated by external factors, many ROS are produced, causing several diseases [16]. DCFH−DA is a sensitive and convenient fluorescent probe that can be used to detect ROS in living cells. The higher the fluorescence, the more ROS accumulate and the more severe the degree of oxidative damage. The results are shown in Figure 6. Compared with the control group, the ROS level in the model group increased to 62.72%. After using the positive control drug vitamin C, the ROS level decreased to 16.58%. After using PPP, ROS decreased compared with the model group. The LG, MG, and HG decreased to 33.49%, 20.2%, and 17.47%, respectively.

### 2.4. The Effect of PPP on Antioxidant Enzyme Activity and MDA Content

**Figure 7:**
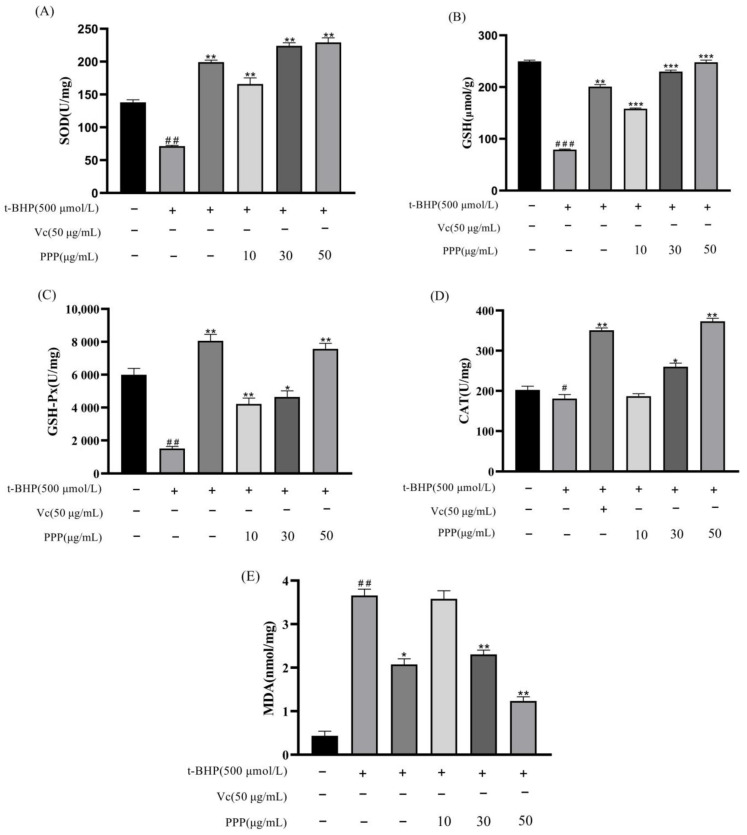
Effect of PPP on SOD activity (**A**), GSH activity (**B**), GSH-Px activity (**C**), CAT activity (**D**), and MDA content (**E**) in HepG2 cells injured by t-BHP. All data are expressed as the mean ± SD according to at least three independent experiments. Compared with the control group, ^#^ *p* < 0.05, ^##^ *p* < 0.01, ^###^ *p* < 0.001; compared with the model group, * *p* < 0.05, ** *p* < 0.05, *** *p* < 0.05.

The levels of intracellular SOD, GSH, GSH-Px, CAT, and MDA can reflect the level of antioxidant activity. Figure 7A−E shows that after t-BHP-induced oxidative damage, intracellular SOD, GSH, GSH-Px, and CAT levels decreased, whereas MDA levels increased, indicating oxidative damage to the cells. After PPP pretreatment, the recovery effect of each indicator level correlated positively with the concentration, and the HG had better recovery effects on GSH and MDA than the Vc group. These results jointly indicated that the protective effect of PPP against t-BHP-induced oxidative stress was primarily attributed to the enhanced activities of CAT, SOD, GSH, and GSH-Px and reduced MDA production in HepG2 cells. The HG showed the strongest antioxidant effect.

### 2.5. Effect of PPP on the mRNA Expression of Antioxidant Enzyme Genes Induced by t-BHP

**Figure 8:**
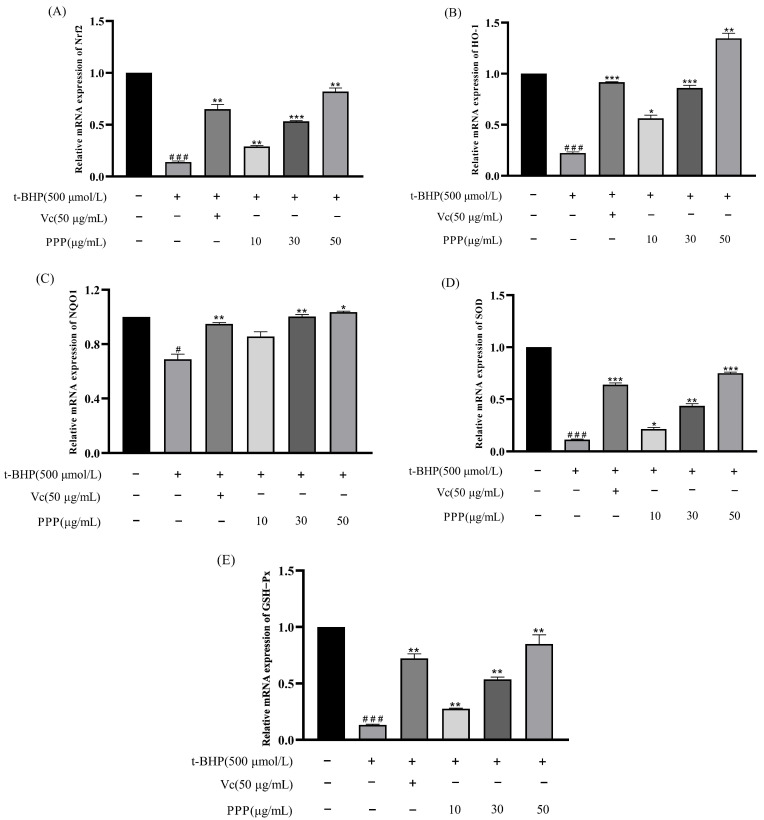
Effects of PPP on the expression of antioxidant-related genes in HepG2 cells under control and oxidative stress conditions. (**A**) Nrf2; (**B**) HO−1; (**C**) NQO1; (**D**) SOD; (**E**) GSH-Px. Values are presented as the mean ± SD (*n* = 3). Compared with the control group, ^#^ *p* < 0.05, ^###^ *p* < 0.001; compared with the model group, * *p* < 0.05, ** *p* < 0.05, *** *p* < 0.05.

Figure 8A–E shows the effects of PPP on the expression of antioxidant-related genes in HepG2 cells. The levels of intracellular SOD, GSH, GSH-Px, MDA, and CAT can reflect the level of antioxidant activity. Compared with the control group, after adding t-BHP to the cells, the expression levels of Nrf2, HO−1, NQO1, SOD, and GSH-Px were significantly reduced, whereas the expression levels of antioxidant-related genes in the Vc and PPP groups were significantly higher than those in the model group. These results indicated that PPP inhibited oxidative stress by upregulating the expression of genes encoding antioxidant enzymes, and the effect correlated positively with the dose. When the concentration reached 50 µg/mL, the expression level of related genes was the highest, and the effect was close to, or even exceeded, that of the Vc group.

### 2.6. Western Blotting

Under normal physiological conditions, Nrf2 mainly binds to its inhibitor Keap1 and is inactive, maintaining a low transcriptional activity. When the ROS in the body rises to a certain level, Nrf2 and Keap1 are uncoupled and activated by stimulation. Keap1 and Nrf2 enter the cell-activated Nrf2/Keap1 signaling pathway. The activated signaling pathway initiates the expression of multiple downstream target proteins. These activated target proteins can regulate the redox balance in the body, allowing the body to recover from oxidative stress to a normal physiological state.

**Figure 9:**
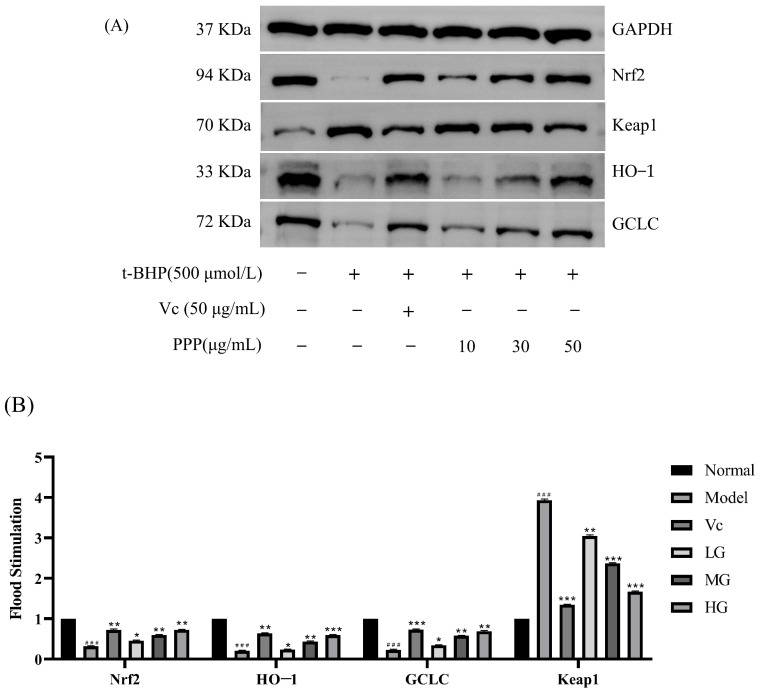
Effects of PPP on the protein expression of the Nrf2/Keap1 signaling pathway in HepG2 cells. (**A**) Protein strip; (**B**) related proteins/-actin relative density. Values are presented as the mean ± SD (*n* = 3). Compared with the control group, ^###^ *p* < 0.001; compared with the model group, * *p* < 0.05, ** *p* < 0.05, *** *p* < 0.05.

As shown in Figure 9, the results indicated that the protein abundance of Nrf2, HO−1, and GCLC significantly decreased after treatment with 500 µg/mL t-BHP for 2 h compared with the control group. In contrast, the protein abundance of Keap1 increased significantly. However, the protein expression of Nrf2 and antioxidant enzymes (HO−1 and GCLC) in HepG2 cells pretreated with PPP and damaged by t-BHP were increased compared with those in the model groups. In contrast, the protein expression of Keap1 was decreased. The increased Nrf2 protein expression and decreased Keap1 protein expression suggest that the Nrf2/Keap1 signaling pathway was activated, and the abundance of HO−1 and GCLC protein expression was consistent with the abundance of Nrf2 protein expression. Comparing the expression levels of various proteins in the HG, MG, and LG, it was found that the high-dose group had the best antioxidant stress effect.

## 3. Materials and Methods

### 3.1. Polygonatum Zanlanscianense Pamp. Stem and Leaf Samples

In mid-March 2022, samples were collected from Group 15, Hongqi Village, Daoming Town, Chongzhou City, Sichuan Province. Associate Professor Long Fei from the Pharmacy Teaching and Research Office of Chengdu University of Traditional Chinese Medicine identified them as the overground part of *Polygonatum zanlanscianense* Pamp. The stems and leaves were separated, washed, dried in a 60 °C oven to constant weight, crushed, and filtered through a fine sieve of 80 µm mesh. The samples were stored in a refrigerator at 20 °C.

### 3.2. Sample Extraction

The crude extract was obtained by concentrating 120 mL of 50% ethanol (Chron Chemicals Co., Ltd., Chengdu, China) extract, which was obtained by extracting 3 g of the sample powder at 50 °C and then ultrasonically assisted for 40 min. This extraction process was repeated three times. The concentration of the extract was carried out at 35 °C under reduced pressure until no alcohol odor was present. The crude extract underwent purification using AB-8 macroporous resin (Huayi Chemical Material Technology Co., Ltd., Shanghai, China) at a sample flow rate of 1.0 mL/min, a sample volume of 20 mL, an eluent concentration of 75%, an elution flow rate of 1.0 mL/min, and an elution volume of 200 mL. The purified eluent was evaporated under reduced pressure at 50 °C until no alcohol taste was observed and freeze-dried in a vacuum freeze dryer (Huayi Chemical Material Technology Co., Ltd., Shanghai, China) at −60 °C for three days to eliminate moisture, and the resulting powdered samples were stored at −20 °C for future use.

### 3.3. Identification and Quantification of Phenolic Compounds

#### 3.3.1. FTIR

According to the solid compression method in the fourth part of the 2020 edition of “The Chinese Pharmacopoeia” [17], the sample PPP was scanned from 4000 to 400 cm^−1^ and the spectrogram was recorded.

#### 3.3.2. UPLC-Q-Obtrip-MS

The chromatographic conditions were as follows: Mobile phase A was a 0.1% formic acid aqueous solution (Chron Chemicals Co., Ltd., China), B was a methanol solution, gradient elution (Chron Chemicals Co., Ltd., China) sequence: 0–20 min, 5%–70% B (*v*/*v*); 20–30 min, 70%–95% B (*v*/*v*). The chromatographic column adopted a Thermo Accucore™ C_18_(100 mm × 3 mm i.d., 2.6 µm) Chromatographic column (Thermo Fisher Scientific, Chengdu, China), with an injection volume of 2 L. The flow rate was 0.4 mL/min, and the column temperature was 28 °C.

The mass spectrum conditions were as follows: An electric spray ion source (ESI) was used. Scan mode: MS/dd MS^2^ positive and negative ion scanning; scanning range *m*/*z* 100–1500; cracking voltage: 3.5 kV (ESI+) and 3.0 kV (ESI−); sheath gas flow rate: 35 arb; auxiliary gas flow rate: 10 arb; ion transfer tube temperature: 320 °C; auxiliary gas temperature: 350 °C; primary resolution: 70,000; secondary resolution: 17,500. In the MS/MS mode, the collision energies in both positive and negative ion modes were set to 20, 40, and 60 eV, respectively.

We imported the raw data collected via UPLC-MS high-resolution mass spectrometry into the Compound Discoverer 3.1 software for peak alignment, peak extraction, and fitting of molecular formulas, and matched fragment ion information results in the mzVault and mzCloud databases to export the matching results. We further analyzed and identified the compounds by referring to the relevant literature and quality deviations.

#### 3.3.3. HPLC-DAD

Chlorogenic acid, caffeic acid, taxifolin, ferulic acid, hyperin, isoquercitrin, rutin, hesperidin, astragalin, quercetin, luteolin, and diosmetin (Desite Biotechnology Co., Ltd., Chengdu, China) were each weighed to 20 mg and dissolved in methanol to form a single standard solution with a mass concentration of 0.5 mg/mL. Then, each, with a concentration of 120 L, was used to form a mixed standard of 12 substances, which were filtered through a 0.22 µm microporous membrane filter. The sample PPP was similarly prepared.

The sample and the mixed standard were analyzed using an HPLC-DAD platform, separated on an Agilent C18 column (250 mm × 4.6 mm, 5 µm). The temperature of the column was maintained at 28 °C, while the flow rate was fixed at 0.8 mL/min. For the mobile phase, a combination of A: water containing 0.3% phosphoric acid and B: pure methanol was used. The injection volume was 10 L, the detection wavelength was 254 nm, and the gradient elution sequence was 0–25 min, 5%–50% B; 25–35 min, 50%–55% B; 35–45 min, 5%–57% B; and 45–60 min, 57%–80% B.

### 3.4. Cell Culture

HepG2 cells were kindly provided by Procell LifeScience & Technology Co., Ltd. in Wuhan in China. The cells were cultured in a basic medium containing 10% fetal bovine serum, 1% double antibody (Beijing Institute of Biological Products Co., Ltd., Beijing, China), and MEM (Beijing Institute of Biological Products Co., Ltd., Beijing, China) at 37 °C with 5% CO_2_ in a cell incubator (Wuhan Servicebio Technology Co., Ltd., Wuhan, China). When cells grew to approximately 80% of the bottle wall, they were digested and passaged with trypsin every 3–4 days. HepG2 cells in a good growth state and logarithmic growth phase were subjected to 5 × 104 per well, with 100 L inoculated into a 96-well plate and incubated at 37 °C or 24 h.

### 3.5. Cell Viability Assay

To determine cell viability, the Cell Counting Kit-8 (Elabscience Biotechnology Co., Ltd., Beijing, China) was utilized. HepG2 cells were exposed to a basic culture medium containing various concentrations of PPP. The PPP concentrations in the wells were 5, 10, 20, 30, 40, 50, 60, 80, and 100 µg/mL. After a 24 h incubation period, 10 µL of CCK-8 solution per 100 µL of medium was added following the manufacturer’s instructions. The cells were then incubated in a 37 °C cell incubator for 2 h. The absorbance was measured at 450 nm using a microplate reader.

HepG2 cells were cultured in a basic medium for 24 h, after which different concentrations of t-BHP (50, 100, 200, 300, 400, 500, 600, 800, and 1000 µg/mL) were introduced. After a 2 h induction period, 10 µL of CCK-8 solution per 100 µL of medium was added according to the manufacturer’s instructions. The cells were incubated in a 37 °C cell incubator for an additional 2 h. The absorbance was subsequently detected at 450 nm using a microplate reader.

In this article, cell viability is characterized by cell survival rate, and the calculation method is shown in Equation (1):

Cell survival rate (%) = (OD _experimental group_ − OD _blank group_)/(OD _control group_ − OD _blank group_) (1)

### 3.6. Intracellular ROS Assay

HepG2 cells with 5 × 104 cells per well in a 96-well plate for 24 h. Control group: an equal amount of basic culture medium was added. Model group: after adding an equal amount of basic culture medium for 24 h, 500 µmol/L t-BHP induction was added for 2 h. Vc group: after 24 h of pre-treatment with an equal amount of vitamin C (5 µg/mL), 500 µmol/L t-BHP induction was added for 2 h. PPP group: after pre-treatment with PPP of three different concentrations (high, medium, and low) for 24 h, 500 µmol/L t-BHP induction was added for 2 h. Cells in each group were cultivated and induced with oxidative stress. Post cultivation, they underwent a washing process with PBS (Wuhan Servicebio Technology Co., Ltd., China), followed by incubation with a DCFH-DA fluorescent probe at a final concentration of 10 µmol/L for 20 min. After three washes with serum-free cell culture medium, the cells were subjected to flow cytometry (CytoFLEX, Beckman coulter, Brea, CA, USA) analysis over time.

### 3.7. Detection of Cellular Antioxidant Enzyme Activity and Malondialdehyde Content

To evaluate the oxidative stress status and antioxidant defense system in the cellular homogenate, a comprehensive analysis was conducted. Specifically, the levels of CAT, GSH, GSH-Px, SOD, and MDA were quantified using assay kits obtained from Wuhan Elabscience (Wuhan, China). To accurately measure the protein concentration in the sample, a BCA protein assay kit provided by Guangzhou Biosharp (Guangzhou, China) was employed.

### 3.8. RNA Extraction and Quantitative Real-Time polymerase Chain Reaction (qRT-PCR)

In accordance with the culturing methodology detailed in Section 2.5, total RNA was isolated from the cells using the RNA pure total RNA rapid extraction kit manufactured by Chengdu Foregene (Chengdu, China). The 5× All-In-One MasterMix (with AccuRT Genomic DNA Removal kit) (Biocompete Inc., Shanghai, China) was used for reverse transcription to obtain cDNA, and qRT-PCR analysis was performed using the EvaGreen Express 2 × qPCR MasterMix-No Dye (Biocompete Inc., Shanghai, China). To accurately quantify the expression level of the target gene, it was normalized to the housekeeping gene GAPDH using the 2^−ΔΔCt^ method. To ensure reliability and precision, each sample was evaluated in triplicate. The primers used are shown in Table 3.

### 3.9. Western Blotting

Total cell proteins were extracted using RIPA cell lysate (Biosharp, Shanghai, China) with a protease inhibitor PMSF (Biosharp, China). Nuclear proteins were obtained using the nuclear and cytoplasmic protein extraction method from the kit (Biosharp, Shanghai, China). Then, they were incubated at 4 °C and centrifuged at 10,000 r/min for 5 min. The supernatant was obtained, and the protein concentration was measured using a BCA reagent kit (Biosharp, Shanghai, China). The concentration of the separation gel was selected according to the molecular weight of the target protein. First, electrophoresis at a constant pressure of 80 V was performed until the bromophenol blue indicator was linear at the junction of the concentrated and separation gels, and then changed to a constant pressure of 120 V until the bromophenol blue reached the bottom of the gel, which took approximately 90 min. After electrophoresis, the membrane was transferred and sealed in TBST containing 5% skimmed milk powder (Guangzhou Saiguo Biotech Co., Ltd., Guangzhou, China) for 2 h. The first antibody (Histone H3 Monoclonal Antibody, ImmunoWay Biotechnology Co., Ltd., Beijing, China) was incubated overnight at 4 °C, and the second antibody (Goat Anti-Rabbit IgG(H+L) HRP, MultiSciences Biotechnology Co., Ltd., Hangzhou, China) was incubated at room temperature for 2 h. ECL (Biosharp, China) was developed and photographed using GAPDH (Affinity, Shanghai, China) as the internal reference.

### 3.10. Statistical Analysis

The test data were statistically analyzed using SPSS 20.0 software, and one-way analysis of variance (ANOVA) and Duncan’s multiple comparisons were used to evaluate significance. All experiments were repeated three times except where stated otherwise, and the results are expressed as the mean ± standard deviation (SD).

## 4. Discussion

Phenolic compounds are an important material basis for the bioactivity of *Polygonatum zanlanscianense* Pamp. stems and leaves. The chemical components of PPP were isolated and identified using infrared spectroscopy, ultra-high-performance liquid chromatography (UPLC), and high-resolution mass spectrometry, with reference to the information in the mass spectrometry database. To the best of our knowledge, this is the first time that 20 phenolic compounds have been identified from *Polygonatum zanlanscianense* Pamp. stems and leaves, and the main components were quantified. Previous studies have extracted the phenolic constituents from the whole herb of *Polygonatum sibiricum* Red. with different solvents of increasing polarity, and it was found that the phenolic constituents of *Polygonatum sibiricum* Red. have strong antioxidant properties, and their bioactivities correlate positively with the total phenolic content [18]. Other authors have previously identified chlorogenic, ferulic, rutin, and ursolic acids as important constituents of *Polygonatum sibiricum* Red. leaves [19].

Excessive ROS cause oxidative stress in cells, thus disrupting the original oxidative– metabolic balance of cells, whereas endogenous and exogenous antioxidants can scavenge excessive ROS and maintain cellular redox homeostasis [20]. Previous studies have shown that hyperoside can inhibit H_2_O_2_-induced oxidative stress injury in LO_2_ cells via the Nrf2/Bach1 antioxidant signaling pathway [21]. Similarly, astragalin, kaempferol, ferulic acid, quercetin, and diosmetin, as natural flavonoids, showed strong free radical scavenging ability in vitro [22,23,24,25]. Caffeic acid passing through the Akt/GSK3β/ Nrf2 pathway reduced oxidative stress damage after cerebral ischemia in rats [26,27]. Chlorogenic acid attenuated doxorubicin-induced oxidative stress in cardiomyocytes via Nrf2/HO-1. New findings showed that the antioxidant activity of hesperidin was not only limited to its radical scavenging activity, but it augmented the antioxidant cellular defenses via the ERK/Nrf2 signaling pathway as well [28]. Rutin enhanced cellular antioxidant activities via the Nrf2/HO-1 signaling pathway [29]. As mentioned above, PPP is mainly composed of these phenolic compounds. Therefore, the inhibition of t-BHP-induced ROS overproduction may be mainly attributed to various phenolic compounds. Subsequently, the effects of PPP on antioxidant enzyme activities and MDA content in the cells were determined, and the results showed that PPP increased SOD, CAT, GSH, and GSH-Px activities and decreased MDA content. The results of this study are consistent with the inhibitory effects of other phenolic compounds on oxidative stress. For example, peony petal flavonoid extract reduced H2O2-induced cellular damage, ROS accumulation, and malondialdehyde content and increased antioxidant enzyme activities in BRL3A cells [30]. Additionally, betel palm polyphenolic extract enhanced the Nrf2 and HO-1 expression in oxidatively damaged cells and reduced ROS production in lipopolysaccharide-induced RAW264.7 cells [31].

Under normal physiological conditions, Nrf2 and Keap1 are localized in the cytoplasm as dimers. Once externally stimulated, Nrf2 dissociates from Keap1 and transfers to the nucleus, where it interacts with the ARE to activate the expression of a series of downstream antioxidant enzymes [32]. In this study, we demonstrated that the expression of the Nrf2 transcription factor was significantly upregulated at both gene and protein levels under PPP intervention, which promoted the translocation of Nrf2 into the nucleus and increased its accumulation in the nucleus, thus improving the antioxidant defense capacity of cells. It was shown that the extract of polymethoxy flavonoid components of citrus reticulata Blanco peels could exert antioxidant activities by activating the Nrf2 signaling pathway, which is consistent with the results of this study [33]. Furthermore, PPP significantly enhanced the cytoprotective proteins HO-1, NQO1, SOD, GSH-Px, and GCLC at both the gene and protein levels and downregulated MDA expression. It can be seen that PPP alleviates oxidative stress damage in cells and exerts antioxidant activity by promoting the transfer of Nrf2 into the nucleus, activating the expression of six downstream antioxidant enzymes of the Nrf2-ARE antioxidant signaling pathway, namely NQO1, SOD, CAT, GSH, GCLC, and GSH-Px, at both the mRNA and protein levels, and downregulates the expression of the stress response proteins, MDA, and Keap1.

The HepG2 cell line has good stability and can be cultured in the laboratory for passages while maintaining stable biological characteristics, ensuring the reliability of experimental results. HepG2 cells have certain metabolic activities and can complete normal physiological metabolic processes, including fat metabolism, oxidative stress, and other processes. Therefore, this study used the HepG2 cell model. However, the use of HepG2 cells to evaluate the protective effect of plant phenolic compounds has a major limitation, which is its low universality. HepG2 cells are cancer cells with different characteristics from normal cells. Therefore, using these cells to evaluate the protective effect of polyphenols may not accurately reflect the effect on normal cells. In addition, HepG2 cells are mainly used for liver cancer research and may not be suitable for studying other types of cells or tissues. Future research should be undertaken to verify whether this protective effect applies to other types of liver cells or normal cells.

## 5. Conclusions

Twenty phenolic compounds were identified from PPP, including three phenylpropanoids, namely caffeic acid, chlorogenic acid, and ferulic acid, and seventeen flavonoids and their glycosides, including kaempferol, dihydroquercetin, kaempferol-3-O-rutoside, fiscone, hesperidin, hyperin, isoquercetin, rutin, quercetin, epicatechin, quercetin, geranium lignin, luteolin, 3-Methoxy-5,7,3, 4-tetrahydroxyflavone, rhododendron, dalspinosin, and genistein B. Among them, chrysoeriol glycoside, zingiberoside, and geranylgeranyl were the main phenolic constituents with higher contents. This study is the first to investigate the composition and application of the stems and leaves of *Polygonatum zanlanscianense* Pamp. The results of cellular experiments showed that PPP had cytoprotective and antioxidant effects, protecting HepG2 cells from t-BHP-induced oxidative stress by scavenging ROS formation and activating the expression of downstream antioxidant enzyme genes of the Nrf2/Keap1 signaling pathway. These findings provide new insights into the mechanism of antioxidative stress in the stems and leaves of *Polygonatum zanlanscianense* Pamp., and offer new ideas for the development of natural antioxidants.

## Figures and Tables

**Table 1:** Phenolic compounds identified in PPP.

No.	Rt/Min	Compounds	Molecular Formula	Additive Ion	Exact Mass	Theoretical Mass	ppm	Characteristic Fragmentation Ions
1	0.87	Caffeic acid	C_9_*H*_8_*O*_4_	[M-H]^−^	180.0352	180.0350	1.11	135.0443
2	6.89	Chlorogenic acid	C_16_*H*_18_*O*_9_	[M-H]^−^	354.0874	354.1708	1.13	191.0191
3	8.45	Kaempferol	C_15_*H*_10_*O*_6_	[M+H]^+^	286.0408	286.0405	1.05	153.0181– 121.0284
4	9.13	Ferulic acid	C_10_*H*_10_*O*_4_	[M-H]^−^	194.0504	194.0504	−1.03	178.0548– 134.9392
5	9.39	Taxifolin	C_15_*H*_12_*O*_7_	[M-H]^−^	304.0512	304.051	0.66	285.0406– 151.0393– 107.0235
6	9.64	Kaempferol-3-O-rutinoside	C_27_*H*_30_*O*_15_	[M-H]^−^	594.1518	594.1511	1.18	285.0326
7	10.15	Fisetin	C_15_H_10_O_6_	[M+H]^+^	286.0555	286.055	1.75	213.0544– 137.0230– 121.0287
8	10.16	Hesperidin	C_28_H_34_O_15_	[M-H]^−^	610.1825	610.1823	−0.33	489.1451– 301.0275
9	10.45	Hyperin	C_21_H_20_O_12_	[M-H]^−^	464.0887	464.0881	1.29	301.0275– 271.0248– 179.0055– 151.0344
10	10.08	Isoquercitrin	C_21_H_20_O_12_	[M-H]^−^	464.0882	464.0882	0	300.0275– 271.0248– 255.0298
11	10.98	Rutin	C_27_H_30_O_16_	[M-H]^−^	610.1467	610.1461	0.98	300.0277– 271.0247– 255.0299
12	12.19	Astragalin	C_21_H_20_O_11_	[M-H]^−^	448.0933	448.0930	−0.67	285.0408– 151.0029– 107.0125
13	12.30	Epicatechin	C_15_H_14_O_6_	[M-H]^−^	290.0714	290.0718	−1.38	245.0816– 203.0722– 123.0465
14	13.37	Quercetin	C_15_H_10_O_7_	[M-H]^−^	302.0359	302.0354	1.66	151.0024
15	13.84	Diosmetin	C_16_H_12_O_6_	[M-H]^−^	300.0567	300.0561	2.00	284.0326– 151.0029
16	15.02	Luteolin	C_15_H_10_O_6_	[M-H]^−^	286.0407	286.0404	1.05	257.0444– 151.0029– 133.0287
17	15.43	3-Methoxy-5,7,3′,4′-tetrahydroxy-flavone	C_16_H_12_O_7_	[M-H]^−^	316.0512	316.051	0.63	300.0276– 283.0535– 283.0535–
18	18.8	Farrerol	C_17_H_16_O_5_	[M-H]^−^	299.0929	299.0925	1.33	271.1834– 179.0342
19	19.16	Dalspinosin	C_18_H_16_O_7_	[M+H]^+^	345.0962	345.0969	−2.03	316.1031– 179.0423
20	25.24	Gardenin B	C_19_H_18_O_7_	[M-H]^−^	360.1129	360.1125	1.11	344.0543– 329.0305– 326.0883– 298.0556

**Table 2:** Species and monomeric phenol contents of PPP.

Peak	Compounds	Regression Equation	R^2^	Content (mg/g)
5	hyperin	y = 410.1x + 0.185	0.9999	76.6
9	astragalin	y = 633.29x + 0.1012	0.9999	21.4
12	diosmetin	y = 507.27x − 0.0038	0.9999	26.4

**Table 3:** The primer sequences for quantitative real-time PCR.

Genes	Primer Sequences
GAPDH	forward: GGAGCGAGATCCCTCCAAAA reverse: GGCTGTTGTCATACTTCTCATGG
Nrf2	forward: AGTCCAGAAGCCAAACTGACAGAAG reverse: GGAGAGGATGCTGCTGAAGGAATC
HO-1	forward: TGCCAGTGCCACCAAGTTCAAG reverse: TGTTGAGCAGGAACGCAGTCTTG
NQO1	forward: GGATGGGAGGTGGTGGAGTCG reverse: AATATCACAAGGTCTGCGGCTTCC
SOD	forward: AGCAGATGACTTGGGCAAAGGTG reverse: ACCACAAGCCAAACGACTTCCAG
GSH-Px	forward: GCAACCAGTTTGGGCATCAGGAG reverse: GCACCGTTCACCTCGCACTTC

## Data Availability

All the data are included in the paper.
